# P62 accumulates through neuroanatomical circuits in response to tauopathy propagation

**DOI:** 10.1186/s40478-021-01280-w

**Published:** 2021-11-02

**Authors:** François-Xavier Blaudin de Thé, Benjamin Lassus, Ari W. Schaler, Stephanie L. Fowler, Chris N. Goulbourne, Ross Jeggo, Clotilde Mannoury la Cour, Mark J. Millan, Karen E. Duff

**Affiliations:** 1 Taub Institute for Research On Alzheimer’s Disease and the Aging Brain, 630 W 168th St, NY 10032 New York, USA; 2 Neuroscience and Immunoinflammation Therapeutic Area, Institut de Recherche Servier, 125 Chemin de Ronde, 78290 Croissy-sur-Seine, France; 3 UK Dementia Research Institute at UCL, University College London, 90 Gower St, W1T 7NF London, UK; 4 HiFiBiO Therapeutics Pépinière Paris Santé Cochin, 29 Rue du Faubourg Saint-Jacques, 75014 Paris, France; 5 UCLA – Physiology Department, 10833 Le Conte Ave, CHS76200, CA 90095 Los Angeles, USA; 6 Nathan Kline Institute: Center for Dementia Research, 140 Old Orangeburg Road, NY 10962 Orangeburg, USA; 7 Present Address: Institute of Neuroscience and Psychology, College of Medicine, Vet and Life Science, Glasgow University, Glasgow, G12 8QQ United Kingdom

**Keywords:** Tauopathy, Spread, p62, Clearance, Microfluidics, Electron microscopy

## Abstract

In Alzheimer’s disease and related tauopathies, trans-synaptic transfer and accumulation of pathological tau from donor to recipient neurons is thought to contribute to disease progression, but the underlying mechanisms are poorly understood. Using complementary in vivo and in vitro models, we examined the relationship between these two processes and neuronal clearance. Accumulation of p62 (a marker of defective protein clearance) correlated with pathological tau accumulation in two mouse models of tauopathy spread; Entorhinal Cortex-tau (EC-Tau) mice where tau pathology progresses in time from EC to other brain regions, and PS19 mice injected with tau seeds. In both models and in several brain regions, p62 colocalized with human tau in a pathological conformation (MC1 antibody). In EC-Tau mice, p62 accumulated before overt tau pathology had developed and was associated with the presence of aggregation-competent tau seeds identified using a FRET-based assay. Furthermore, p62 accumulated in the cytoplasm of neurons in the dentate gyrus of EC-Tau mice prior to the appearance of MC1 positive tauopathy. However, MC1 positive tau was shown to be present at the synapse and to colocalize with p62 as shown by immuno electron microscopy. In vitro, p62 colocalized with tau inclusions in two primary cortical neuron models of tau pathology. In a three-chamber microfluidic device containing neurons overexpressing fluorescent tau, seeding of tau in the donor chamber led to tau pathology spread and p62 accumulation in both the donor and the recipient chamber. Overall, these data are in accordance with the hypothesis that the accumulation and trans-synaptic spread of pathological tau disrupts clearance mechanisms, preceding the appearance of obvious tau aggregation. A vicious cycle of tau accumulation and clearance deficit would be expected to feed-forward and exacerbate disease progression across neuronal circuits in human tauopathies.

**Supplementary Information:**

The online version contains supplementary material available at 10.1186/s40478-021-01280-w.

## Introduction

Despite their clinical heterogeneity, neurodegenerative diseases are characterized by the accumulation of pathological neurotoxic proteins within neurons and/or glia [64]. Hyperphosphorylated, conformationally abnormal tau protein accumulates in several neurodegenerative diseases, collectively known as tauopathies [28, 72]. This diverse group of diseases includes Alzheimer’s disease (AD), frontotemporal lobe dementia (FTD), progressive supranuclear palsy (PSP), argyrophilic grain disease (AGD) and Pick’s disease (PiD), which vary in terms of the brain regions and cell type affected as well as the precise species of tau that accumulates [37]. Post mortem mapping studies have shown that in AD, 3-repeat (3R) and 4-repeat (4R) tau pathology spreads in a stereotypic manner from the transentorhinal cortex to anatomically connected regions of the hippocampus and then to neocortical association areas [33, 34]. Recently, these findings have been validated in patients using PET tracers against tau [18]. Despite being less well described and affecting a different set of regions, similar disease progression can be seen in other tauopathies [18], including PSP [42, 74], AGD [78] and PiD [38].

Several hypotheses have been proposed to explain how AD pathology evolves. One hypothesis is that specific brain regions, or classes of neuron [24, 25] have differing intrinsic susceptibility to the development of tau pathology [71], with some structures affected earlier and others later in the disease. However, more recently it has been proposed that seeds (aggregation-competent monomers or oligomers of pathological proteins) can physically spread across neuronal connections via synapses and induce pathology in connected (recipient) neurons via a templating mechanism, similar to what is observed with prion proteins [3, 51]. Several mouse models have been generated to test the ‘spread’ hypothesis. One approach involves the injection of pathological tau seeds from different sources into the brains of tau transgenic or wildtype (WT) mice, which leads to enhanced or de novo pathology respectively, first at the injection locus, and later in remote, but connected regions [7, 8, 12, 29, 36, 39, 53]. A second approach examines the spread of endogenous tau in tau transgenic mouse lines that express mutant human tau at high levels in disease-relevant brain regions (for example the superficial layers of the entorhinal cortex (EC) and the subiculum in the EC-tau line) [6, 45]. Tau pathology that develops in these regions spreads to anatomically connected regions of the hippocampus, and at later stages to the perirhinal and frontal cortices [23], recapitulating the progressive spread of human tau pathology in AD at early Braak stages (I-III). The hypothesis that the spread of tauopathy between neurons could be mediated by the passage of tau seeds across the extracellular space was inferred from in vivo studies [6, 19] and strengthened by in vitro studies using neurons that were engineered to develop tau pathology or stimulated to do so by the addition of exogenous tau seeds [5, 12, 68, 75].

One factor that potentially contributes to the accumulation and spread of misfolded or aggregated proteins in neurodegenerative diseases is defective intracellular clearance by the ubiquitin proteasome system (UPS) and/or the autophagy-lysosomal network (ALN) [2, 28]. Macro-autophagy, herein referred to as autophagy, is a cellular process that engulfs cytoplasmic content and transports it to the lysosomes for degradation [57]. Mutations in genes related to both UPS and ALN pathways of clearance are found in several neurodegenerative diseases including Parkinson’s disease and amyotrophic lateral sclerosis, and to a lesser extent AD [14, 15, 50]. Correspondingly, markers of defective clearance such as autophagosome accumulation and reduced proteasomal clearance are found in the brains of patients with AD [58]. Moreover, pathological tau has been shown to inhibit both autophagy and the UPS, reinforcing the significance of neurotoxic proteins in tauopathies and other neurodegenerative diseases [2, 31, 52, 44, 57, 58]. SQSTM1 (p62), a cargo protein which binds to autophagy substrates and is degraded with them [49, 59] colocalizes with tau and α-synuclein aggregates in the brain of patients with various neurodegenerative diseases including AD, PSP, Lewy body disease and multiple system atrophy [69]. The accumulation of p62 is therefore a sign that cargos destined to be cleared by autophagy have been insufficiently degraded and is widely used as a marker of protein clearance dysfunction, in particular autophagy, both in patient tissues and animal models [41, 69, 76]. Additional markers of clearance dysfunction can be found in many animal models of neurodegenerative diseases, and activation of either the UPS or the ALN has been shown to ameliorate pathology in these models [21, 52, 55, 60, 61, 73].

To study the link between tau spread and clearance, pathological tau and p62 accumulation was measured in two in vivo models of tau spread; PS19 mice injected with seeds and EC-Tau mice [6, 45]. In both models, p62 accumulation preceded overt tau pathology as established using the conformational antibody MC1, but correlated with tau seed accumulation, suggesting that they could be potent modulators of clearance pathways. Interestingly, immuno electron microscopy (iEM) of synapses showed that p62 and MC1 colocalized at the point of tau spread. In vitro, p62 and pathological tau were shown to colocalize in two primary neuronal culture models. Significantly, in an in vitro microfluidic network containing cortical primary neurons, pathological tau accumulation and transfer negatively impacted clearance mechanisms in both donor and recipient neurons, implying that the propagation of pathological tau can drive clearance dysfunction.

## Material and methods

### Culture, seeding, and harvest of HEK293 CFP/YFP biosensors

The HEK293 CFP/YFP biosensor cells stably express the repeat domain of tau (TauRD) fused to either CFP or YFP in the same cell line. When the cells are exposed to brain material containing pathogenic tau proteins, the CFP/YFP reporters aggregate, allowing for the generation of a Fluorescence Resonance Energy Transfert (FRET) signal. All protocols were undertaken as described previously [26], with only minor changes. Briefly, 24 h prior to seeding, the biosensors were plated into wells of a flat-bottomed 96-well plate at 30 000 cells per well in a volume of 130 µL of regular serum-containing culture media. The cells were allowed to settle undisturbed for 15 min at RT prior to being moved to the incubator. The next day, seeding mixes were made in 20 µL volumes × 6 (n of 5 plus 1 extra) in a separate 96-well plate (treatment plate). Each 20 µL seeding reaction contained 15 µL protein dilution (10 µg protein sample in a final volume of 15 µL Optimem media), and 5 µL Lipofectamine dilution (1 µL lipofectamine plus 4 µL Optimem media). Seeding reactions were incubated for 30 min in the cell culture incubator, and 20 µL was added gently to each well of cells 24 h post plating (cells were ~ 65–70% confluent). The seeded cells were maintained undisturbed for 48 h prior to harvest for FRET measurement.

At time of harvest, the cell media was removed from each well using a vacuum manifold for 96-well plates. Trypsin–EDTA (50 µL) was added to each well, and the plate was incubated for 2–3 min at 37 °C until cells were lifted from the plates. Serum-containing media (150 µL) was added to each well, the cells were triturated 10X each, and moved into wells of a fresh v-bottomed 96-well plate (v-bottom plate significantly improved cell recovery and the reproducibility of FRET measurements). Samples in v-bottom plates were centrifuged at 600 × g for 5 min, and the media supernatants were removed. Resultant cell pellets were resuspended thoroughly by triturating 10 × in 150 µL cold 4% paraformaldehyde (PFA) in PBS. Cells were left to fix for 10 min, and re-centrifuged at 600 × g for 5 min. The PFA supernatants were removed, and the cells were carefully and completely resuspended in 200 µL PBS + 1 mM EDTA.

### FRET flow cytometry assessment of tau seeding

Resuspended cells were immediately analyzed on a BD LSR Fortessa flow cytometer equipped with a high throughput sampler, according to the parameters previously described [26]. Samples were run on the “low” flow setting (< 1 000 events/sec), and 50 000 events were captured per well. The mean % FRET^+^ cells from 5 replicate wells for each sample.

### Animals

Protocols and procedures were approved by the Committee on the Ethics of Animal Experiments of Columbia University and according to Guide for the Care and Use of Laboratory Animals of the National Institutes of Health. We used PS19 tau transgenic mice of both genders (n = 8) which express human tau (1N4R) with the P301S mutation found in patients with familial FTLD-tau [77]. Animals were anesthetized using ketamine/xylazine, placed in a stereotaxic frame and injections were made into the Dentate Gyrus of both hemispheres (DS9 – left, DS1 – right) (bregma -2.5 mm, lateral ± 2.0 mm, depth − 2.4 mm) using a Hamilton syringe. A total of 15 µg of biosensor HEK (overexpressing TauRD) lysate were injected at a rate of 0.2 µl/min. DS1 and DS9 HEK lysate were collected as described previously [12, 39]. EC-Tau mice which overexpress human 4R tau with the P301L mutation in the outer layers of the medial EC were generated and studied previously in the lab [23, 45]. Mice were anesthetized with ketamine and xylazine and transcardially perfused with PBS then 10% formalin, and post-fixed in 10% formalin overnight at 4 °C. Brains were incubated overnight in PBS + 30% sucrose at 4 °C and frozen in OCT compound (Fischer 4585). Brains were cut horizontally (35 µm) in a cryostat and kept at 4 °C in PBS + 0.02% Azide. For primary cultures we used SAS Sprague Dawley Rat purchased from Charles River or E14 PS19 mice bred in the animal facility at Columbia University.

### Immunohistochemistry

For fluorescent staining, slices were washed with PBS and blocked for 1 h with PBS + 0.3% triton X-100 + 5% Normal Goat Serum (NGS) (Vector S-1000). Slices were then incubated with the primary antibody in the blocking solution at 4 °C overnight, then washed 3 times in PBS + 0.1% Triton X-100. Slices were incubated in the secondary antibody in the blocking solution for 1 h at room temperature and washed the same way. Slices were mounted on microscopy slides (Fisherbrand 22-037-246) and incubated with Sudan Black (Sigma - 199,664) (0,3% w/v in 70% Ethanol) for 5 min to block auto–fluorescence then washed 2 × 5 min with 70% Ethanol and 3 × 5 min with PBS + 0.02% Tween20. Slides were mounted with fluoromount DAPI (ThermoFischer -00-4959-52).

For DAB staining slices were immersed in PBS + 10% H_2_0_2_ + 10% Methanol to block endogenous peroxidases and washed 2 × with PBS. The primary and biotinylated secondary antibody steps are the same as for fluorescence. Slices were then incubated in streptavidin-HRP (Vector PK-6100) except for MC1, where the secondary antibody was directly linked to HRP; finally, the HRP developed with DAB (Vector SK-4100). Slices were then washed 3 × with PBS and mounted on microscopy slides. Slides were dried 30 min at 37 °C and dehydrated in Ethanol (70% for 4 min, 95% for 4 min, 100% for 2 × 5 min) and in xylene (2 × 10 min) and finally mounted in DPX medium (Sigma—06,522). The stained sections were inspected by light microscopy (Olympus BX50, Olympus) for DAB staining and on a confocal microscope for fluorescence (Zeiss). The number of positive neurons for MC1 and p62 was calculated manually on three brain slices per animal, using the software ImageJ for the following regions: hippocampus subdivided into the DG, CA3, CA1 and subiculum as well as for the entorhinal, perirhinal and frontal cortices.

For in vitro, cultures were fixed in 4% paraformaldehyde (PFA), 4% sucrose for 15 min at room temperature. Cells were then washed twice with PBS for 5 min and permeabilized for 10 min with 0.2% Triton X-100 and then 1% of goat serum in PBS for the saturation step. Primary antibodies were then added, and the samples incubated at 4 °C overnight in PBS. The samples were rinsed twice for 5 min with PBS and further incubated with the corresponding secondary antibodies for 2 h at room temperature. Coverslips were then mounted on a microscopy slide with mounted media. The chips were then rinsed once with PBS and once with PBS + 0.1% sodium-azide.

The following primary antibodies were used: MC1 1/500 (gift of Peter Davies), bassoon 1/500 (Abcam, ab82958), p62 1/500 (Abnova, M012C11), Tubulin 1/1000 (Sigma T5168), Map2 1/500 (Sigma, M4403), PSD95 1/50 (ThermoFischer 51–6900). Species-specific secondary antibodies coupled to Alexa 350, 405, 488, 555, or 633 were used (1/500, Invitrogen) to visualize bound primary antibodies.

### Thioflavin S staining

For Thioflavin S treatment slices were washed with PBS and blocked for 1 h with PBS + 0.3% triton X-100 + 5% NGS and washed with PBS. Fluorescent immunochemistry and Sudan Black treatment were done as previously described. Slides were incubated in Thioflavin S (1% in distilled water) for 8 min at room temperature. Slides were washed 2 × 3 min in 80% ethanol and 3 min 95% ethanol and then 3 × with PBS. Slides were finally mounted in fluoromount DAPI.

### Statistical analysis

Statistical analysis was done using the software Prism. For all analyses, the type of test, parameters and statistical results are described either in the results part or in the supplementary information (Additional file 6).

### Electron microscopy

For EM, mice were anesthetized as previously described and transcardially perfused directly with the ice-cold EM fixative solution (0.1 M Sodium Cacodylate + 2% glutaraldehyde + 4% PFA). Brains were then dissected and kept in fixative at 4 °C. Brains were sectioned using the vibratome into 50 μm coronal sections. Sections were left in fixative at 4 °C for at least 24 h. Samples were then treated with 1% osmium tetroxide for 1 h, washed in distilled water four times (10 min/wash), and then treated with 2% aqueous uranyl acetate overnight at 4 °C in the dark. Samples were washed and sequentially dehydrated with increasing concentrations of ethanol (20, 30, 50, 70, 90 and 100%) for 30 min each, followed by three additional treatments with 100% ethanol for 20 min each. Samples were then infiltrated with increasing concentrations of Spurr's resin (25% for 1 h, 50% for 1 h, 75% for 1 h, 100% for 1 h, 100% overnight at room temperature), and then incubated overnight at 70 °C in a resin mold. Sections of 70 nm were cut on a Leica ultramicrotome with a diamond knife. The sections were placed on to carbon formvar 75 mesh nickel grids and etched using 4% sodium metaperidotate for 10 min before being washed twice in distilled water and then blocked for one hour. Grids were incubated with P62 and MC1 antibodies (both at 1 in 10 dilution) at 4 °C overnight. Next day grids underwent seven washes in 1xPBS and were then incubated in anti-rabbit 6 nm and anti-mouse 10 nm gold secondary (both at 1 in 50 dilution) for 1 h. After this the grid was washed seven times in 1xPBS and twice in distilled water. Grids were then post stained in 1% uranyl acetate for 5 min followed by two washes in water and then stained with lead citrate for 5 min followed by a final two washes in water. Samples were then imaged on a ThermoFisher Talos L120C operating at 120 kV. Negative controls for iEM included leaving out the primary antibodies and incubating with just the secondary.

### Microfluidic chamber production

The microfluidic devices were composed of large channels (50 µm in height) for cell injection and thin channels (3 µm in height) for axon growth. For the template, two layers of photoresist were used (SU82050 and SU82002). The three-compartment chips were composed of three parallel rectangular macro-channels separated by 2 arrays of microchannels. Neurons were seeded in the left and right chamber (length: 2000 µm; width: 500 µm, height: 60 µm) and were able to connect in the middle chamber (length: 2000 µm; width: 300 µm, height: 60 µm). The left and the middle cell culture chambers were separated by a long array of microchannels (length 500 µm, height 3 µm) only axons were able to cross. The second and the third chamber were separated by a short array of microchannels (length 50 µm, height 3 µm) that dendrites were able to cross. Polydimethylsiloxane (PDMS) (Sylgard 184) was mixed with a curing agent (9:1 ratio) and degassed. The resulting preparation was poured onto the microfluidic mold and reticulated at 70 °C for 2 h. The elastomeric polymer print was detached, and 2 reservoirs were punched for each macro-channel (6 reservoirs in total). The resulting piece was cleaned with isopropanol and dried. The polymer print and a glass coverslip were treated for 180 s in an air plasma generator (98% power, 0.6 mBar, Diener ZEPTO) and bonded together. Chips were placed under UV for 30 min and then coated with a solution of poly-D-lysine (10 µg/ml) overnight and washed with PBS before cell seeding.

### Primary neuronal cultures

Cortices were micro-dissected from 5 SAS Sprague Dawley Rat embryos or 7 to 10 E14 embryos of PS19 mice. All steps of the dissection were performed in cold Gey’s balanced salt solution (GBSS) supplemented with 0.1% glucose. Dissected structures were digested with papain (20U/ML in Neurobasal, Sigma). After papain inactivation with FBS, structures were mechanically dissociated with a pipette in presence of DNAse. After several rounds of rinsing, cells were re-suspended in Neurobasal + B27 in a final density of 35 million cells/mL. For 24 well plate, 150 000 to 200 000 of cortical precursors are plated. For microfluidic cultures, WT cortical neurons were then seeded in the left and the right chamber: 2 µl of the cell suspension was introduced into the upper reservoir and cells flowed into the chamber and adhered within 20 min. For 24 well plate and for microfluidic devices, cortical neurons are plated in plating media for 24 h: neurobasal + B27 (Gibco) + streptomycin/penicillin (Gibco) + 10% FBS. The next day, a media change is performed to remove the FBS, neurobasal + B27 (Gibco) + streptomycin/penicillin (Gibco). Microfluidic chips were placed in plastic Petri dishes containing H2O-EDTA to prevent evaporation and incubated at 37 °C in a humid 5% C02 atmosphere. The culture medium was renewed every 6 days.

### Harvesting seed-competent material from mouse brain tissue

Tau transgenic mice (line rTg4510) overexpressing human P301L tau were dissected into EC/hippocampus (Hippo), perirhinal cortex (PRh) and frontal cortex (Front) in ice-cold PBS. Tissues were snap frozen in liquid nitrogen and placed at − 80 °C in 1.5 mL Eppendorf tubes overnight. Following freezing, the tissues were thawed on ice, and homogenized until smooth in seeding buffer (50 mM Tris–Cl, pH 7.5 150 mM NaCl + 0.05% Triton-X + Pierce protease inhibitor tablet, EDTA –free [A32965] added just before use, pH 7.4) using sterilized polytetrafluorethylene (PTFE) pestles for 1.5 mL Eppendorf tubes fitted to a battery powered hand-held grinder. Samples were centrifuged at 500 × g for 5 min at 4 °C, and the resultant supernatants were further cleared at 1000 g for 5 min at 4 °C. The final protein supernatants were adjusted to the same total protein content following BCA protein determination.

### Lentiviral transduction and rTg4510 mouse brain lysate incubation

Cortical neurons from a wild type (WT) rat were transduced at DIV1 with lentivirus expressing LM TauRD-YFP (P301L/V337M), (gift of Marc Diamond) [40]. In 24 well plates, the plating media was removed and replaced with 500 µL containing 2 µL of viral suspension at 10^9^ viral particles per ml. In microfluidic devices, the plating media was removed and 10 µL of culture media was added in each reservoir. Then, 2 µL of viral suspension at 10^9^ viral particles per ml was added to one of the reservoirs of each chamber. A 15 µL flow was induced in each chamber to perfuse the virus in the cell culture chamber. The media was not changed at this point. At DIV4, 50 µg of rTg4510 brain lysate or 10 µg of DS9 seeds were added to the 24 well plate or in one reservoir of the donor chamber in the microfluidic chips. WT brain lysate or DS1 seeds were used as negative controls. Cells were incubated for 8 h and then a complete media change was performed.

### Super resolution microscopy

Structured-illumination microscopy and image processing were performed in the Confocal and Specialized Microscopy Shared Resource of the Herbert Irving Comprehensive Cancer Center at Columbia University with a Nikon Ti Eclipse inverted microscope controlled with NIS-Elements. The platform is supported by a NIH/NCI Cancer Center Support Grant P30CA013696. The structured illumination microscope was purchased with NIH grant #S10 OD014584. Neurons were transduced in 24 well plates with a SAP102-GFP lentivirus (gift from Clarissa Waites, CUMC) as described in the lentiviral transduction part for the LM TauRD-YFP lentivirus transduction. The SAP102-GFP lentiviral suspension was 10^9^ viral particles per ml. Fluorescence excitation: 405 nm for MAP2, 488 nm for Bassoon, PSD95 or SAP102-GFP, and 561 nm for MC1. Antibodies and immunochemistry protocols are described under’ immunohistochemistry’.

## Results

### Clearance dysfunction is associated with propagated tau pathology in vivo

**Fig. 1:**
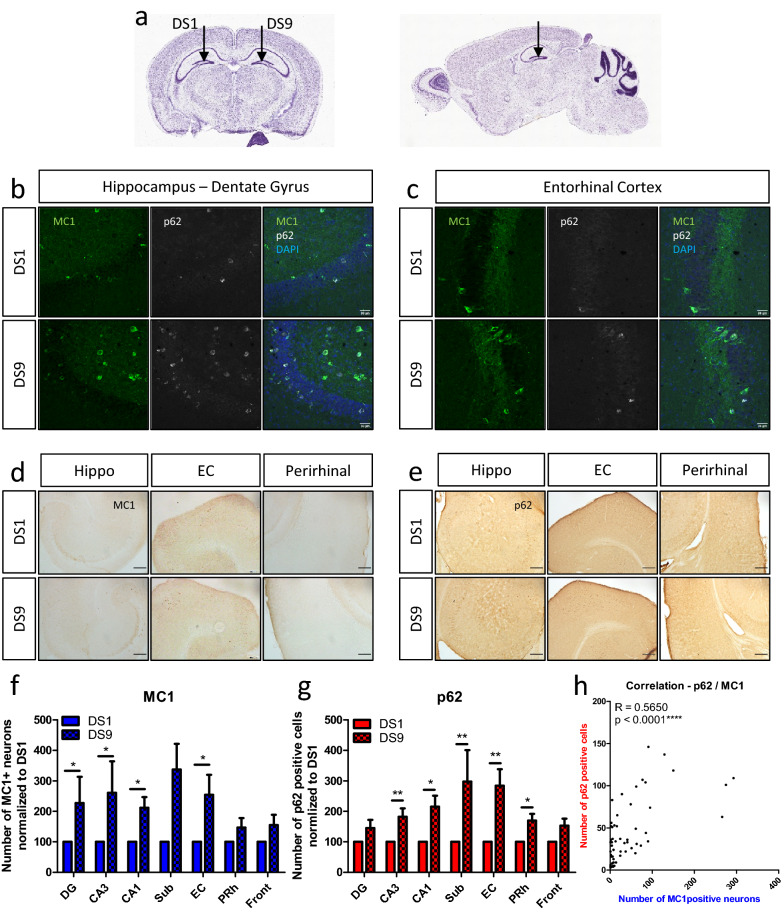
Tau transfer and clearance dysfunction in the seeded PS19 mouse. **a** DS9 (tau competent seeds) and DS1 (control) injections on each side of the dentate gyrus in the same PS19 mouse. Image source: Allen brain atlas. **b**–**c** Immunofluorescence images: MC1 (tau with a pathological conformation—green), p62 (marker of ubiquitinated cargos destined to degradation—white) and DAPI (nuclei—blue) staining on PS19 mice on the DS1 injected side (upper panel) and DS9 (lower panel) for the dentate gyrus. (**b**) and the entorhinal cortex (**c**). Scale bar represents 30 μm. **d**–**e** DAB staining of the hippocampus, entorhinal and perirhinal cortices of PS19 mice on the DS1 injected side (upper panel) and DS9 (lower panel) for MC1 (**d**) and p62 (**e**). Scale bar represents 200 μm. **f**–**g** Quantification of the number of positive neurons is shown for MC1 (**f**) and p62 (**g**) for the dentate gyrus, CA1, CA3, subiculum and the entorhinal, perirhinal and frontal cortices. Data is normalized to the DS1 side, paired t-test for each region. **h** Correlation between the number of MC1 and p62 positive neurons in each injected PS19 mouse and for each measured region, on both the DS1 and the DS9 side

To model tau spread in PS19 mice which express human tau with the P301S mutation [77], tau seeds (derived from the HEK cell biosensor line [12, 35]) were inoculated into the hippocampal dentate gyrus (DG) and left for 30 days. Mice were injected on one side with the pro-aggregating tau seed DS9 and on the other side with DS1, a similar but seeding-incompetent form of tau that allowed for a within-mouse control (Fig. 1a). In these mice a striking co-localization was seen between p62 and pathological tau immuno-labeled with the conformational antibody MC1, in the DG and in the connected EC [11] (Fig. 1b–c). MC1 was significantly increased in the DS9 compared to DS1 injected side (Fig. 1d) for the DG (t_(7)_ = 2.5, p = 0.041, paired t-test). MC1 staining was also increased in the rest of the hippocampus (CA3 and CA1) and in the EC (Fig. 1f) confirming that tau pathology formation and the spread of pathology had been accelerated by DS9 seeds. The p62 staining was also increased (Fig. 1e) on the DS9 compared to DS1 sides, in the CA3, CA1 as well as in the entorhinal and perirhinal (PRh) cortices (Fig. 1g). In addition, there was a statistically significant correlation between p62 and MC1 levels in these areas (Fig. 1h) (see supplementary information for detailed statistics).

**Fig. 2:**
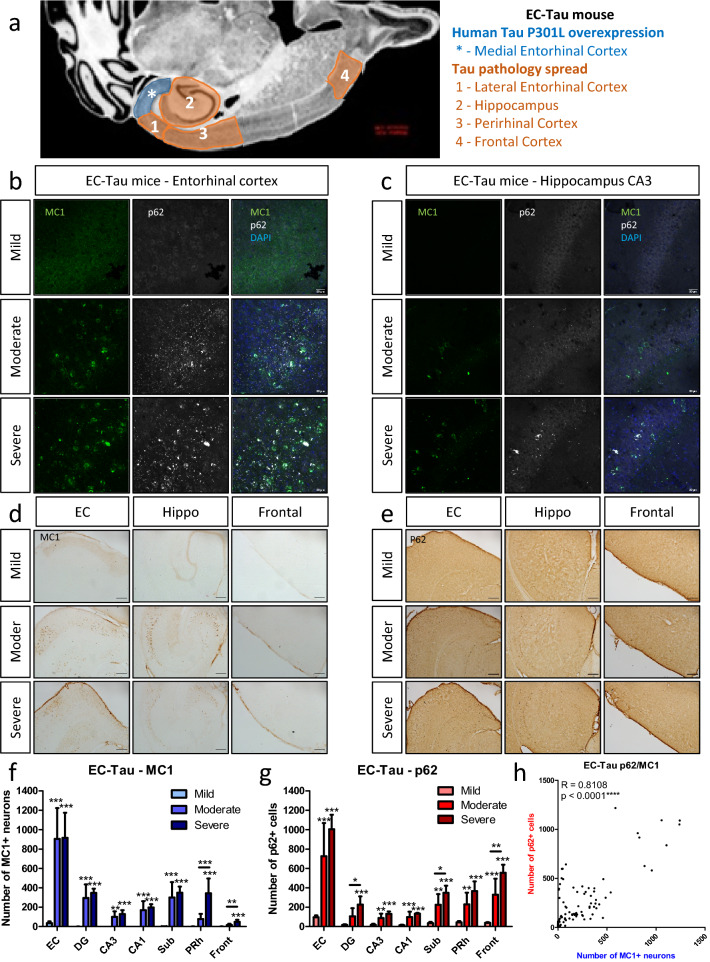
Tau transfer and clearance dysfunction in the EC-Tau mouse. **a** Tau pathology spread in the EC-Tau Mouse from the lateral EC to the frontal cortex. Image source: Allen brain atlas. **b**–**c** Immunofluorescence images: MC1 (green), p62 (white) and DAPI (blue) staining in EC-Tau mice with mild (upper panel), moderate (middle panel) and severe (bottom panel) pathology for the EC (**b**) and the CA3 (**c**). Scale bar represents 30 μm. **d**–**e** DAB staining of the hippocampus, entorhinal and frontal cortices on EC-Tau mice with mild (upper panel), moderate (middle panel) and severe (bottom panel) pathology for MC1 (**d**) and p62 (**e**). Scale bar represents 200 μm. **f**–**g** Quantification of the number of positive neurons is shown for MC1 (**f**) and p62 (**g**) for the dentate gyrus, CA1, CA3, subiculum and the entorhinal, perirhinal and frontal cortices. Absolute numbers, one-way ANOVA with post-hoc test for each region. **h** Correlation between the number of MC1 and p62 positive neurons in the EC-Tau mice and for each measured region

In the EC-Tau mouse model of endogenous pathological tau spread, human P301L mutant tau is expressed at high levels in the pre- and para subiculum, the outer layers (layer II/III) of the medial EC, and to a lesser extent, the lateral EC [6, 45]. As previously reported [6, 23, 45], in old EC-Tau mice, MC1-positive tau accumulated in the EC, in the hippocampus and in remote territories of the neocortex, like the frontal cortex (Fig. 2a) indicating spread of tau pathology. Similar to PS19 mice, a striking co-localization was seen between MC1 and p62 in the EC, where the pathology originates (Fig. 2b) and also in secondary regions of spread like CA3 (Fig. 2c). The distribution of the pathology in these mice was used to define three stages: mild (7–10 months), where MC1 positive tau was restricted to the EC; moderate (17–22 months), where pathological tau had spread to the hippocampus, and severe (29–31 months), where tau pathology was widespread, including the PRh and frontal cortices (Fig. 2d). Co-localization of MCI-positive tau and p62 was observed (Fig. 2b–c) and a detailed analysis was performed in multiple regions of the brain. Quantification validated the regional increase of MC1 positive neurons at the different stages in the EC, as well as in all the other regions studied (Fig. 2f) (see supplementary information for detailed statistics). Similarly, in all the regions studied, p62 increased with MC1-positive pathology (Fig. 2g) (see supplementary information for detailed statistics) and there was a striking correlation between these two markers (R = 0.8108, *p *< 0.0001, Spearman correlation) (Fig. 2h). In addition, some p62 also co-localized with ubiquitin (Additional file 1: Fig. 1a) and partially with the proteasome-specific K48-ubiquitin (Additional file 1: Fig. 1b). Furthermore, a triple co-localization study between MC1, p62 and thioflavin-S, a marker of β-sheet containing proteins, showed that p62 was co-localized not only with mature tau aggregates but also with thioflavin-S negative tau, suggesting p62 accumulates before different tau conformers amass into mature aggregates (Additional file 1: Fig. 1c).

### p62 accumulates before overt tau pathology in EC-Tau mice

**Fig. 3:**
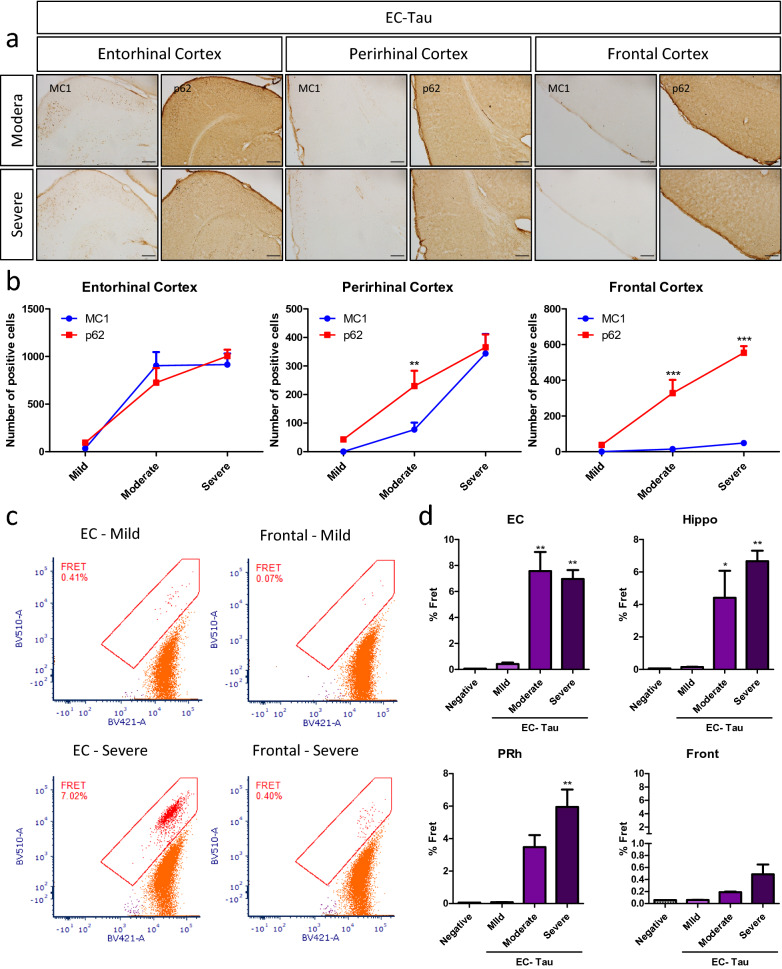
P62 accumulation precedes MC1 positive tau pathology in the EC-Tau mouse. **a** DAB staining of the entorhinal, perirhinal and frontal cortices on EC-Tau mice with moderate (upper panel) and severe pathology (bottom panel) for MC1 (left panel) and p62 (right panel). Scale bar represents 200 μm. **b** Quantification of the number of positive neurons for MC1 and p62 is shown in EC-Tau mice with mild, moderate and severe pathology for the entorhinal, perirhinal and frontal cortices. Absolute numbers, two-way ANOVA with post-hoc test. **c** FACS sorting of Tau biosensor HEK cells treated with brain lysate from the entorhinal (left panel) and frontal cortices (right panel) of EC-Tau mice with mild (top panel) and severe pathology (bottom panel). **d** Quantification of the seeding ability (% FRET in the biosensor cells) for the hippocampus and entorhinal, perirhinal and frontal cortices of EC-Tau mice with mild, moderate and severe pathology. One-way ANOVA with post-hoc test

As tau pathology development in the EC-Tau mouse can be predicted and mapped both temporally and spatially, we were able to examine the relationship between pathological tau and p62 as the pathology propagated through the brain during the three stages of pathology; mild, moderate and severe. Surprisingly, extensive p62 staining was observed in the perirhinal and frontal cortices at a relatively early stage, before somatodendritic tau had accumulated (Fig. 3a); p62 staining was assessed in age-matched WT littermates, but no cytoplasmic accumulation similar to that of EC-Tau mice was seen in any of the mice (data not shown).

Quantification of markers confirmed these observations for the PRh cortex in the EC-Tau mice at the moderate pathology stage, and for the frontal cortex in mice at both moderate and severe pathology stages (Fig. 3b). One explanation for this is that p62 might accumulate with tau species such as soluble monomers or oligomers that are diffusely distributed or were poorly detected by the MC1 antibody, which recognizes a disease-related conformational change in the tau protein. To test whether tau present in these areas was nonetheless competent to seed tau aggregation, brain lysate from PRh and frontal cortex was applied to a FRET based tau aggregation assay [26]. Data showed that the regions without overt tau pathology nevertheless contained aggregation-inducing tau species (see supplementary information for detailed description and statistics) (Fig. 3c–d).

**Fig. 4:**
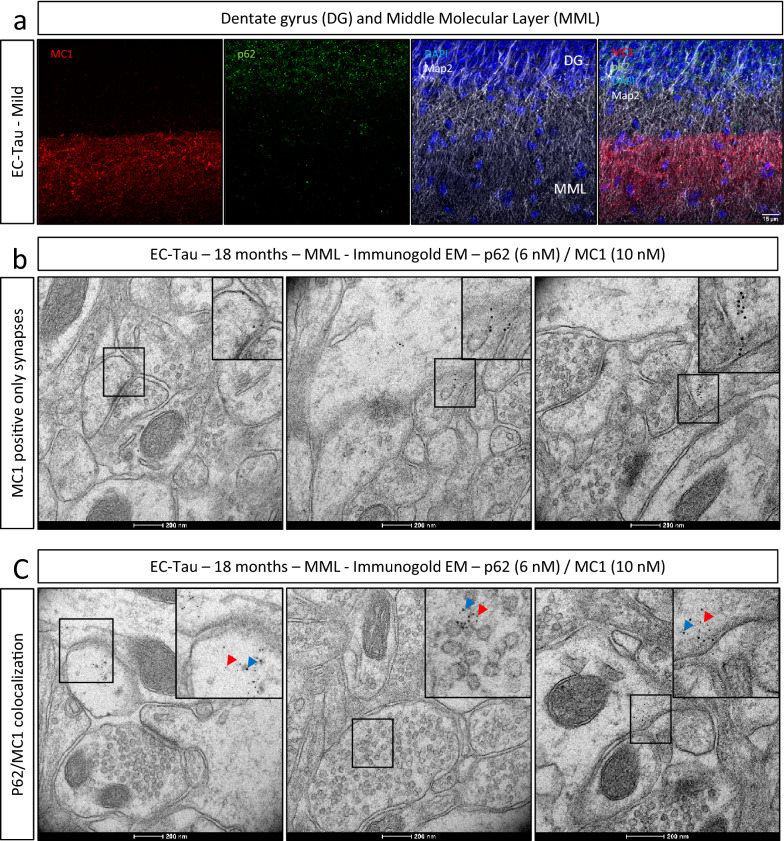
Tau and p62 subcellular localization in the EC-Tau mouse dentate gyrus. **a** Low magnification immunohistochemistry image of the middle molecular layer (MML) of an EC-Tau mouse with low pathology (restricted to the EC): conformational abnormal human tau was labelled with antibody MC1 (red). Image shows co-labelling with an antibody against p62 (green) and DAPI labelled nuclei (blue). Scale bar represents 15 μm. **b** Representative immuno electron microcopy (iEM) images of synapses in the middle molecular layer (MML) of an 18 month old EC-Tau mouse double labelled with p62 (6 nm gold beads) and MC1 (10 nm gold beads). Image shows tau pathology (MC1 antibody) only. Scale bar represents 200 nm. **c** Representative iEM images of the colocalization of p62 (red arrow) and MC1 (blue arrow) in the middle molecular layer (MML) of an 18 month old EC-Tau mouse double labelled with p62 (6 nm gold beads) and MC1 (10 nm gold beads). Scale bar represents 200 nm.

We also assessed EC-Tau mice with mild pathology. At this stage, MC1-positive tau only accumulates in neurons located in layer II/III of the medial EC and is also present in their axons and presynaptic axon terminals that terminate in the middle molecular layer (MML) of the DG. Axons in the MML from synapses with dendrites from granule cells located in the granule cell layer of the DG. At this stage, MC1-positive tau is restricted to axons in the MML and is not detectable in granule cell dendrites or cell bodies. Triple labeling with MC1, p62 and the dendrite marker Map2 showed that while MC1-positive tau was abundant in axons as expected, p62 was most abundant in the granule cell layer, identified by DAPI stained nuclei (Fig. 4a). As pathological tau is thought to spread across the MML trans-synaptically, we were interested in whether MC1-positive tau was associated with clearance dysfunction within synapses and organelles in this region. Light microscopy immunofluorescence showed a few spots of p62 immunolabeling in the MML (Fig. 4a). To better visualize the structures involved, mild to moderate stage EC-Tau mice were analyzed by iEM. MC1-positive tau was observed in pre- and post-synaptic compartments synapses (Fig. 4b) and for a several synapses in this layer, a striking colocalization was seen between MC1 and p62 (Fig. 4c). These data suggest that tau seed accumulation in the synapse, in the earliest stages of cell-to-cell spread, is associated with dysfunction of clearance mechanisms likely contributing to trans-synaptic propagation.

### Neuronal p62 accumulation precedes microgliosis and neuronal loss

To assess whether tauopathy induced p62 accumulation was associated with microgliosis and neuronal loss, EC-Tau mice were stained with Cd68 (a marker of phagocytic microglia) and NeuN (a marker of neuronal nuclei) and compared to age-matched WT littermates to control for the effects of aging, independent of tau pathology. Counts of NeuN positive nuclei in EC-Tau mice indicated overt neuronal loss in the para-subiculum in mice at the moderate stage, and in the medial EC in severe stages, but not in other regions. Microgliosis was also seen in the EC at both stages, and in the hippocampus of mice with severe pathology. The appearance of these markers occurred later than that of p62, suggesting that microglia-derived inflammation and pathogenicity were not likely to be causative of p62 accumulation (Additional file 2: Fig. 2a–f).

### Pathological tau colocalizes with p62 in vitro in primary neurons and is sensitive to proteasome inhibition

**Fig. 5:**
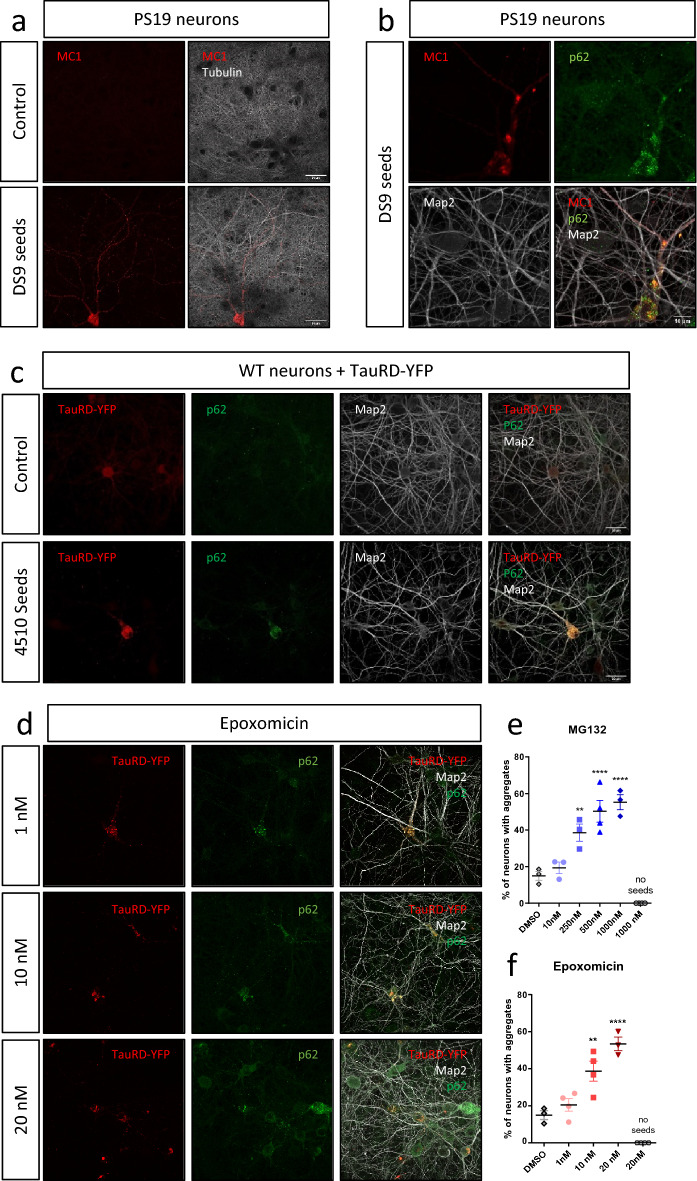
Tau pathology and clearance deficits in two primary neuron models of tauopathy. **a** Immunofluorescence images of MC1 (pathological tau—red) and α-tubulin (microtubules—white) in DIV 15 (days of culture) cortical neurons from PS19 mice (overexpressing human tau P301S) exposed to DS1 seeds (upper panel) or to DS9 seeds (lower panel). Scale bar represents 30 μm. **b** Co-localization between MC1 (red) and p62 (green) in DIV 15 neuronal dendrites (labelled with Map2, white) from PS19 cortical neurons exposed to DS9 seeds. Scale bar represents 10 μm. **c** Rat neurons treated with a lentivirus overexpressing the tau repeat domain tagged with yellow fluorescent protein (TauRD-YFP) showing immunofluorescence images of tau tagged with YFP (red), p62 (green) and Map2 (white) in DIV 18 cortical neurons exposed to WT mouse brain lysate (upper panel) or rTg4510 (overexpressing human tau P301L) brain lysate (lower panel). Notice the aggregation of TauRD-YFP in the presence of pathological brain lysate. Scale bar represents 30 μm. **d**–**f** Images showing that increasing doses of pharmacological inhibitors of the proteasome—epoxomicin (1 to 20 nM) or MG132 (10 nM to 1 µM)—triggered an increase of the percentage of neurons with TauRD-YFP inclusions. Images are shown for epoxomicin. Scale bar represents 30 μm  (**e**); quantification is shown for both (**e**–**f**)

To further examine the relationship between pathological tau spread and p62 accumulation, both processes were studied in primary neuron models. To induce the formation of pathological tau, cortical neurons from PS19 embryos were exposed to either aggregation inducing DS9 tau seed or non-aggregating DS1 as a control. After DIV18, MC1-positive tau was detected in PS19 neurons exposed to DS9 seeds, but not to DS1 (Fig. 5a). In DS9 seeded neurons, MC1 co-localized with p62 in the somatic compartment and in dendrites (Fig. 5b). Similar to in vivo, pathological tau could been seen at the synapse by immunofluorescence and super resolution microscopy (Additional file 3: Fig. 3a). To confirm these results in another model, a viral YFP-tagged tau repeat domain (TauRD-YFP) biosensor construct with the P301L/V337M (LM) mutation was expressed in rat cortical primary neurons. At DIV18, in the presence of brain lysate from WT mice, TauRD-YFP was not seeded and did not form inclusions (Fig. 5c, top panel). However, TauRD-YFP inclusions were seen, mainly in the somatic compartment of neurons, upon application of brain lysates from rTg4510 mice that had robust tau pathology. In this model, there was extensive colocalization between tau inclusions and p62 (Fig. 5c, bottom panel). Here again pathological tau could be found at the synapse (Additional file 3: Fig. 3b). To confirm the role for protein clearance in tau accumulation in this model, increasing doses of proteasome inhibitors were added for two days (Fig. 5d). A concentration-dependent increase in TauRD-YFP inclusions was observed after incubation with both MG132 (F_(5,14)_ = 31.03, *p* < 0.0001) (Fig. 5e) and epoxomicin (F_(4, 13)_ = 32.51, * p*< 0.0001) (Fig. 5f). Interestingly, in the absence of seeds, epoxomicin and MG132 did not trigger de novo TauRD-YFP inclusion formation.

### Pathological tau spread induces p62 accumulation directly in a three-chamber microfluidic network

**Fig. 6:**
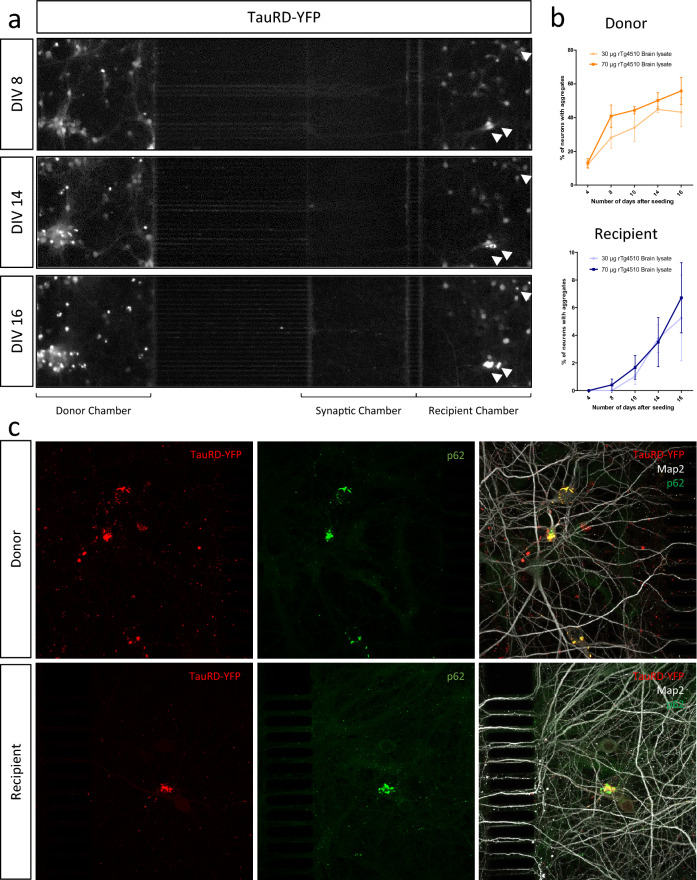
Transsynaptic spread of pathological tau inhibits clearance mechanisms in recipient neurons grown in a microfluidic device. **a** Timecourse study of the spread of TauRD-YFP inclusions in cortico-cortical network reconstructed in microfluidic devices. Rat cortical neurons in the donor (left) chamber connect via their axons to cortical neuron dendrites in the recipient (right) chamber via synaptic connections in the middle chamber. The three panels show the same cortico-cortical network at DIV8, DIV14 and DIV16. White arrows highlight the progressive aggregation of TauRD-YFP in neurons in the recipient chamber. **b** Quantification of progressive TauRD-YFP aggregation in neurons in the donor chamber (top panel) and in the recipient chamber (bottom panel). **c** Confocal immunofluorescence images showing the colocalization between TauRD-YFP aggregates (red), p62 (green) and Map2 (white), in the donor (top panel) and recipient (bottom panel) neuron chambers at DIV 16 in a microfluidic device

To further evaluate whether spread-induced tau pathology drives p62 accumulation, we constructed a neuronal network in a three-chamber microfluidic device. Primary cortical neurons plated in the left (donor) and right (recipient) chamber were separated by a third chamber where axons from the donor neurons connected at a synapse with dendrites (immuno-positive for Map2) from recipient neurons. Both sets of neurons were transduced with the TauRD-YFP expressing lentivirus. To study the impact of tauopathy propagating from donor to recipient neurons, the donor side was incubated with tau seeds from rTg4510 mice. The fluidic isolation of the chambers was ensured by adding a larger volume of media in the middle chamber, thereby preventing passive diffusion of rTg4510 brain lysate between donor and recipient sides. By DIV8, seeding led to TauRD-YFP inclusion formation in the donor compartment but not yet in the recipient chamber at this time-point (Fig. 6a, top panel). In the same microfluidic chamber, by DIV14, inclusions had increased in the donor chamber and TauRD-YFP inclusions could now also be seen in the recipient chamber (Fig. 6a, middle panel, white arrow). At DIV16, 41% and 57% of neurons in the donor side exhibited somatic YFP inclusions after incubation with 30 and 70 μg of rTg4510 brain lysate respectively (Fig. 6b, top panel); in the recipient chamber, YFP inclusions were seen in 8% of neurons for the 70 μg condition (Fig. 6a and b, bottom panels). At this stage, YFP inclusions in both donor and recipient chambers were positive for p62 (Fig. 6c) and pathological tau could be found at the synapse in the recipient chamber (Additional file 3: Fig. 3c). These data indicate that propagated tauopathy has the ability to inhibit intracellular clearance mechanisms across a neuronal network.

## Discussion

In this study, two in vivo models of tau spread were studied; the EC-Tau mouse line and the PS19 mouse line injected with pathological tau seeds. In vitro, tau spread was modelled in a three-chamber microfluidic device with primary cortex neurons grown in the donor and recipient chambers connected via synapses situated in a middle chamber. In all three models, tau pathology spread was accompanied by p62 accumulation.

### Pathological tau spread in both in vivo and in vitro models

In PS19 mice, the injection of seed-competent tau (DS9) into the DG led to an increase of MC1 positive tau pathology compared to the DS1 (control) injected side, not only at the injection locus, but also in several connected regions, namely the CA1 and CA3 and in the EC. In the EC-Tau mouse, the spread of tau pathology via neuronal connections was shown by the presence of MC1 positive tau at the synapses, in both pre and post synaptic compartments, by iEM.

To model the spread of tau pathology in vitro, we used a specific microfluidic device in which rat primary cortical neurons were grown in two chambers separated by a third one where neurons connect via the axons from the first chamber and the dendrites from the second. Microfluidic models of synaptic connections have been instrumental in showing the trans-synaptic spread of pathological proteins, including tau [30, 54] or synuclein [27], and also trans-synaptic degeneration [13]. The use of a viral vector overexpressing YFP-tagged tau allowed us to follow the spread of tau pathology temporally, after seeding with tau seeds within the donor chamber and subsequent spread to the recipient chamber via the connections in the synaptic chamber.

### Tau pathology spread leads to p62 accumulation

In both in vivo and in vitro models, tau pathology spread was accompanied by p62 accumulation. In EC-Tau mice, iEM showed that p62 colocalizes with several autophagy related structures: the Golgi apparatus, autophagosomes and lysosomes (Additional file 4: Fig. 4), illustrating the link between this protein and the autophagy process. P62 helps deliver ubiquitinated cargo for autophagic degradation as part of autophagic flux [41, 56] and as p62 is itself degraded in the process, the accumulation of p62 is widely considered a sign of defective clearance of autophagosomes [1, 41, 49, 56, 59, 76]. The function of p62 in clearance goes beyond the autophagy pathway as p62 is also linked to the UPS, shuttling polyubiquitinated proteins, including tau, to the proteasome for degradation [47, 69]. This autophagy receptor also colocalizes with proteasomes under basal condition as well as upon UPS inhibition [49] and proteasome inhibition has been shown to induce p62 transcription and enhance autophagy [49]. Recent publications also showed that p62 is implicated in the phase separation of ubiquitinated proteins [10, 56, 59], underscoring its relevance as a marker for clearance mechanisms in general, linking the UPS and autophagy [46, 62]. The abnormal accumulation of p62 in all our model systems can therefore be interpreted as indicative of a general dysfunction of neuronal clearance mechanisms. This is supported in our study by the fact that in EC-tau mice, p62 partially colocalized with the proteasome specific K-48 ubiquitin. Additional experiments with more direct readouts could nevertheless confirm the inhibition of autophagy and UPS in these models.

Although markers of clearance pathway dysfunction have been assessed in relation to tau pathology previously, studies have mainly examined the presence of clearance markers in relation to mature tau pathology [22, 32]. Spatio-temporal correlations between mature and nascent pathological forms of tau, and the linear sequence of events linking tau accumulation, tau pathology propagation and protein clearance dysfunction have not been studied in detail. We show that in EC-Tau mice, p62 accumulation is a very early event in the pathological process, and it occurs before the appearance of somatodendritic MC1 positive tau and well before inflammation (Cd68) or neurodegeneration (loss of NeuN) – a sequence of events also seen in AD patients [20, 59, 65, 70]. Strikingly, in both in vivo models, while MC1 tau could be seen in neuronal processes, p62 accumulated in the soma, demonstrating a potential link between local and global clearance mechanisms. Local p62 accumulation could be mediated by the presence of small seed-competent forms of tau as EC-Tau mice showed p62 immunoreactivity in areas such as the perirhinal cortex that were devoid of overt somatic MC1 staining (Fig. 3a), but where seeds could be detected by a sensitive seeding assay. It was also seen in the synapses of the DG where putative MC1 positive tau seed material was shown to colocalize by iEM (Fig. 4c). The p62 positive granule cell bodies seen in the early stage of tauopathy propagation (Fig. 4a) could reflect a response to propagated, undetected tau seeds that have transferred from the pre-synaptic compartment of axons terminating in the MML. But it is also possible that the granule cells are manifesting dysfunctional protein clearance independent of tau seed accumulation, due to the connection of their dendrites to axon terminals in the MML filled with MC1 positive tau. Overall, the data suggests that clearance pathway dysfunction occurs very early in the pathological cascade.

The in vitro model of tau spread in microfluidic devices provided a simplified system to further address the linear relationship between trans-synaptic spread and clearance dysfunction. In this model, p62 accumulation in neurons in the recipient chamber occurred only after tau had spread from the donor chamber, and it colocalized with the newly templated tau aggregates formed from propagated tau seeds. In this model, contrary to the EC-Tau mouse, p62 accumulation did not precede tau accumulation. One difference between the two models lies in the recipient neuronal population. In EC-Tau mice, pathological tau is not overexpressed in the DG and it arises via spread from the donor region—the entorhinal cortex—which occurs slowly and progressively. On the contrary, in the microfluidic device, both the donor and recipient chambers were treated with the lentivirus overexpressing TauRD-YFP making it a more aggressive and faster tau spread model, making the EC-Tau mouse a tau spread model closer what happens in the disease.

In support of our findings, a recent phosphorylated tau interactome saw p62 as a strong interactor and among the two most significantly enriched pathways were proteins belonging to the UPS and those involved in phagosome maturation [17]. The ability of pathological tau to inhibit the UPS has been shown previously in the rTg4510 model of tauopathy, where pathological tau accumulates rapidly [52], and in the brain of AD patients [66]. A similar effect of tau pathology on autophagy was also seen [4, 9, 22, 48]. Finally, our study is in accordance with clinical data, as both p62 accumulation and seeding ability appear before strong tau aggregation in the brain of patients at the earliest stages of Alzheimer’s disease [20, 59, 65, 70].

### A potential vicious circle between tau accumulation and the inhibition of clearance mechanisms

This study is in accordance with the hypothesis that the impairment of clearance mechanisms, namely autophagy and the UPS, is a key mediator of tauopathy. Though further work will be required to better define their respective significance, pharmacological inhibition of proteasomal activity in the primary neuron model with seeded tauopathy led to an increase in aggregated tau accumulation, and similar results have previously been shown for the inhibition of autophagy [43, 63, 67]. However, proteasome inhibition did not induce tau aggregation in the absence of pre-existing seeds, even at the highest dose, suggesting that tau seeds are necessary to induce tau aggregation. Given the ability of pathological tau to further inhibit clearance mechanisms, it is likely that a vicious circle will be triggered once aggregates develop, with serious consequences for the cell [16, 30]. Activators of both clearance pathways have been shown to decrease tau pathology in different animal models [31, 52, 60, 61, 73] which, alongside this study, supports the use of therapeutic approaches that aim to improve clearance pathways in an attempt to prevent or retard tau pathology spread through neural circuits. Tau accumulation and clearance dysfunction are not accompanied by neuronal loss in our in vivo models. However, inflammation and neurodegeneration have been shown in the medial entorhinal cortex of EC-Tau mice with severe pathology, a region where pathological tau accumulation starts. Therefore, tau pathology and clearance dysfunctions could ultimately lead to inflammation and neurodegeneration in the long term, but more studies are required to validate this hypothesis and the intrinsic toxicity of tau pathology is still a debated subject [2, 28].

In conclusion, our observations from two in vivo mouse models and a complementary in vitro microfluidic model suggest that the transfer of small pathological tau species between neurons substantially impairs protein clearance mechanisms, here measured by p62 accumulation, thereby accelerating the accumulation of pathological tau in neuroanatomically connected neuronal networks. This may ultimately lead to the propagation of tau pathology to distant regions of the brain and the spread of neurodegeneration. We propose that tau seeds are transferred from the donor to recipient neuron, where they increase pathological tau via prion-like template seeding and simultaneously impair clearance mechanisms both locally at the synapse and in the whole neuron, although this needs to be further explored with other markers of clearance. These two consequences of tau spread—pathological tau accumulation and inhibition of protein clearance mechanisms—act together in a vicious circle ultimately leading to widespread pathogenicity and neurodegeneration (Additional file 5 Fig. 5). More generally, these observations support the potential therapeutic utility of early reinforcement of UPS, ALN and other clearance mechanisms to impede neurotoxic protein spread and the progression of neurodegenerative disorders.

## Supplementary Information

**Additional file 1. Fig. 1**. P62 co-localizes with ubiquitin and mature tau aggregates in the EC-Tau mouse. (a) Immunofluorescence images: ubiquitin (green), p62 (white) and DAPI (blue) staining in EC-Tau mouse brain with severe pathology in two regions, EC (left panel) and the CA3 (right panel). Scale bar represents 30 μm. (b) Immunofluorescence images: proteasome relevant K-48 ubiquitin (green), p62 (white) and DAPI (blue) staining in EC-Tau mouse brain with severe pathology in two regions, EC (left panel) and the CA3 (right panel). Scale bar represents 30 μm. (c) Immunofluorescence images: MC1 (green), p62 (white) and Thioflavin S (beta-sheets aggregates - red) staining in EC-Tau mouse brain with mild (upper panel), moderate (middle panel) and severe (bottom panel) pathology in the EC region. Scale bar represents 50 μm.

**Additional file 2. Fig. 2**. Neurodegeneration and inflammation in the EC-Tau mice is due to tau pathology and not aging. (a) DAB staining for Cd68 (activated microglia - left panel) and NeuN (neuronal nuclei - right panel) in the EC of EC-Tau mice with severe pathology (top panel) and age matched WT mice (bottom panel). Scale bar represents 200 μm. (b) Quantification of the % of Cd68 positive area (left panel) and normalized NeuN positive area (right panel) in the para-subiculum (PaS) (top panel), medial EC (middle panel) and lateral EC (bottom panel) of EC-Tau mice with mild, moderate and severe pathology, and age matched WT controls. (c) DAB staining for Cd68 (left panel) and NeuN (right panel) in the hippocampus of EC-Tau mice with severe pathology (top panel), and age matched WT mice (bottom panel). Scale bar represents 200 μm. (d) Quantification of the % of Cd68 positive area (left panel) and normalized NeuN positive area (right panel) in the Dentate Gyrus (DG) (top panel), CA3 (top middle panel), CA1 (bottom middle panel) and subiculum (bottom panel) of EC-Tau mice with mild, moderate and severe pathology, and age matched WT controls. (e) DAB staining for Cd68 (left panel) and NeuN (right panel) in the frontal cortex of EC-Tau mice with severe pathology (top panel), and age matched WT mice (bottom panel). Scale bar represents 200 μm. (f) Quantification of the % of Cd68 positive stained area (left panel) and normalized NeuN positive area (right panel) in the perirhinal cortex (top panel) and frontal cortex (bottom panel) of EC-Tau mice with mild, moderate and severe pathology, and age matched WT controls.

**Additional file 3. Fig. 3**. Pathological tau localization at the synapse in all three in vitro models. (a) Immunofluorescence images of MC1 (pathological tau - red), Map2 (neuronal dendrites - white) and different synaptic markers (green): bassoon (pre-synaptic), PSD95 and Sap102-GFP (post-synaptic) in DIV 15 cortical neurons from PS19 mice (overexpressing human tau P301S) exposed to DS9 seeds. Images were taken with a confocal (left panel) or using structured-illumination microscopy. Scale bar represents 10 μm for immunofluorescence. (b) Immunofluorescence images of MC1 (pathological tau - red), Map2 (neuronal dendrites - white) and bassoon (pre-synaptic – green) in rat primary neurons treated with a lentivirus overexpressing TauRD-YFP and rTg4510 tau seeds. Scale bar represents 10 μm. (c) Immunofluorescence images of MC1 (pathological tau - red), Map2 (neuronal dendrites - white) and bassoon (pre-synaptic – green) in rat primary neurons treated with a lentivirus overexpressing TauRD-YFP. Images were taken in the recipient chamber of a microfluidic device after treatment of the donor chamber with tau seeds extracted from rTg4510 mice.

**Additional file 4: Fig. 4**. p62 and MC1 colocalization with autophagy related structures. Images of p62/MC1 immunogold electron microscopy showing the Golgi apparatus (top panel), autophagosomes and lysosomes (bottom panel) all part of the cellular protein clearance system. Scale bar represents 200 μm.

**Additional file 5: Fig. 5**. Proposed model for the link between tau spread and inhibition of clearance mechanisms. (a) Transsynaptic spread of pathological tau from donor to recipient neurons (top) correlates with the spread of clearance deficits (bottom). (b) Pathological templating on native (bottom) tau & inhibition of clearance mechanisms, UPS and autophagy (top). (c) Representation of the proposed vicious circle between pathological tau accumulation and the inhibition of clearance mechanisms

**Additional file 6.** Detailed statistics for quantifications in main and supplementary figures.

## Data Availability

The authors declare that the data supporting the findings of this study are available within the article or its supplementary information file.

## Abbreviations

AD: Alzheimer’s disease
AGD: Argyrophilic grain disease
ALN: Autophagy/lysosome network
ALS: Amyotrophic lateral sclerosis
CFP: Cyan fluorescence protein
DG: Dentate gyrus
DIV: Days in vitro
EC: Entorhinal cortex
EDTA: Ethylenediaminetetraacetic acid
FACS: Fluroescence-activated Cell Sorting
FBS: Fetal bovine syndrome
FRET: Förster resonance energy transfer
FTD: Frontotemporal lobe dementia
Map2: Microtubule associated protein 2
NeuN: Neuronal nuclei antigen
PBS: Phosphate buffer saline
PDMS: Polydimethylsiloxane
PFA: Paraformaldehyde
PiD: Pick’s disease
PRh: Perirhinal
PSP: Progressive supranuclear palsy
PTFE: Polytetrafluorethylene
TauRD: Tau repeat domain
UPS: Ubiquitin proteasome system
WT: Wildtype
YFP: Yellow fluorescence protein
