# Supplementary figures and images for: P62 accumulates through neuroanatomical circuits in response to tauopathy propagation
Source: Acta Neuropathol Commun. 2021 Nov 2;9:177. doi: 10.1186/s40478-021-01280-w (PMC8561893; doi:10.1186/s40478-021-01280-w)

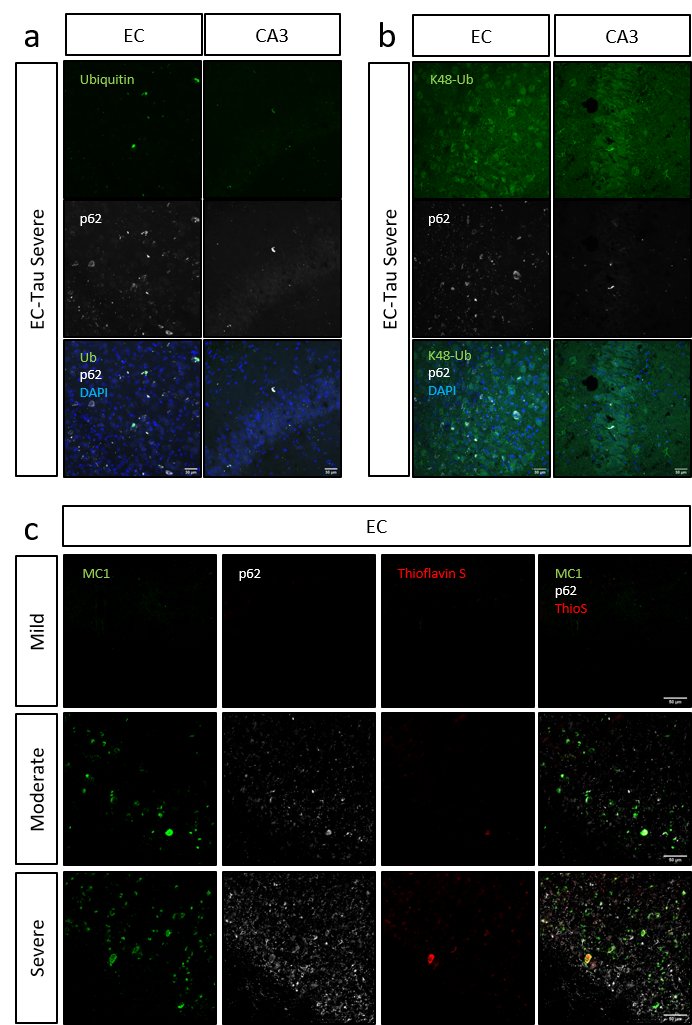

Supplement: Supplementary file 1 — Additional file 1. Fig. 1. P62 co-localizes with ubiquitin and mature tau aggregates in the EC-Tau mouse. (a) Immunofluorescence images: ubiquitin (green), p62 (white) and DAPI (blue) staining in EC-Tau mouse brain with severe pathology in two regions, EC (left panel) and the CA3 (right panel). Scale bar represents 30 μm. (b) Immunofluorescence images: proteasome relevant K-48 ubiquitin (green), p62 (white) and DAPI (blue) staining in EC-Tau mouse brain with severe pathology in two regions, EC (left panel) and the CA3 (right panel). Scale bar represents 30 μm. (c) Immunofluorescence images: MC1 (green), p62 (white) and Thioflavin S (beta-sheets aggregates - red) staining in EC-Tau mouse brain with mild (upper panel), moderate (middle panel) and severe (bottom panel) pathology in the EC region. Scale bar represents 50 μm. [file 40478_2021_1280_MOESM1_ESM.tif]

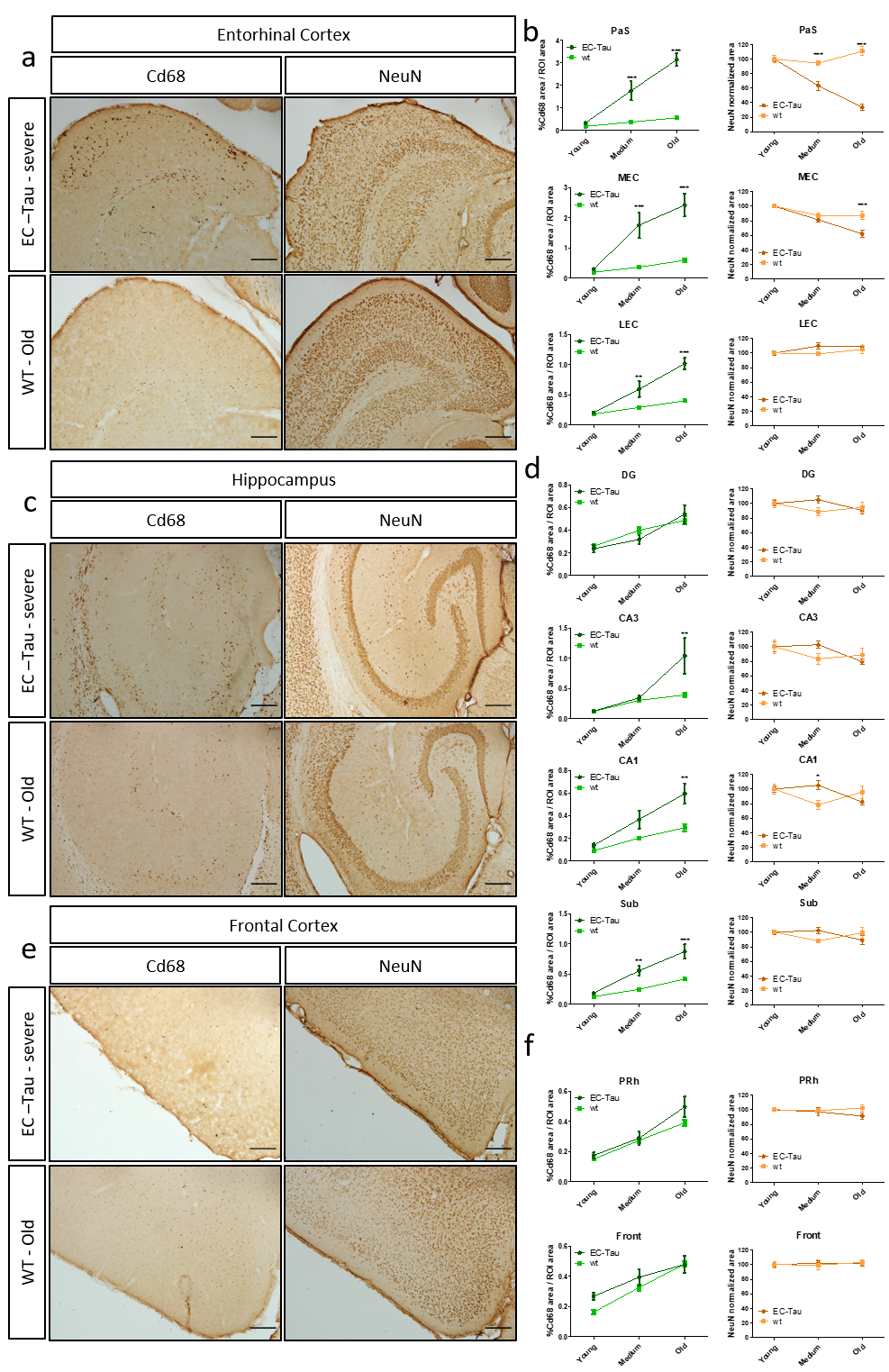

Supplement: Supplementary file 2 — Additional file 2. Fig. 2. Neurodegeneration and inflammation in the EC-Tau mice is due to tau pathology and not aging. (a) DAB staining for Cd68 (activated microglia - left panel) and NeuN (neuronal nuclei - right panel) in the EC of EC-Tau mice with severe pathology (top panel) and age matched WT mice (bottom panel). Scale bar represents 200 μm. (b) Quantification of the % of Cd68 positive area (left panel) and normalized NeuN positive area (right panel) in the para-subiculum (PaS) (top panel), medial EC (middle panel) and lateral EC (bottom panel) of EC-Tau mice with mild, moderate and severe pathology, and age matched WT controls. (c) DAB staining for Cd68 (left panel) and NeuN (right panel) in the hippocampus of EC-Tau mice with severe pathology (top panel), and age matched WT mice (bottom panel). Scale bar represents 200 μm. (d) Quantification of the % of Cd68 positive area (left panel) and normalized NeuN positive area (right panel) in the Dentate Gyrus (DG) (top panel), CA3 (top middle panel), CA1 (bottom middle panel) and subiculum (bottom panel) of EC-Tau mice with mild, moderate and severe pathology, and age matched WT controls. (e) DAB staining for Cd68 (left panel) and NeuN (right panel) in the frontal cortex of EC-Tau mice with severe pathology (top panel), and age matched WT mice (bottom panel). Scale bar represents 200 μm. (f) Quantification of the % of Cd68 positive stained area (left panel) and normalized NeuN positive area (right panel) in the perirhinal cortex (top panel) and frontal cortex (bottom panel) of EC-Tau mice with mild, moderate and severe pathology, and age matched WT controls. [file 40478_2021_1280_MOESM2_ESM.tif]

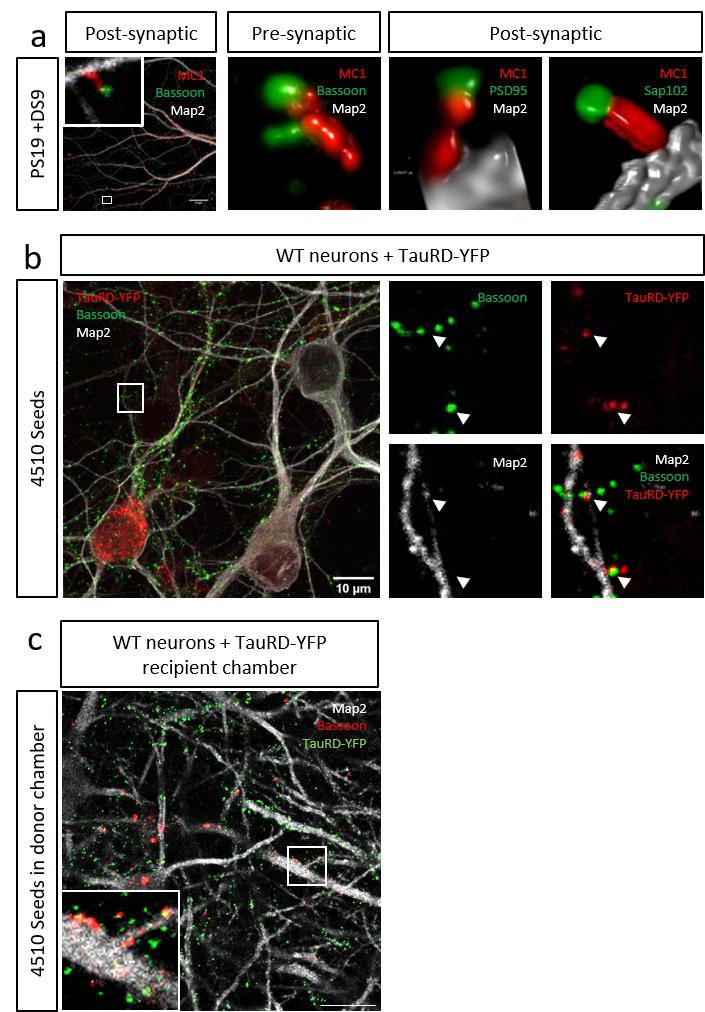

Supplement: Supplementary file 3 — Additional file 3. Fig. 3. Pathological tau localization at the synapse in all three in vitro models. (a) Immunofluorescence images of MC1 (pathological tau - red), Map2 (neuronal dendrites - white) and different synaptic markers (green): bassoon (pre-synaptic), PSD95 and Sap102-GFP (post-synaptic) in DIV 15 cortical neurons from PS19 mice (overexpressing human tau P301S) exposed to DS9 seeds. Images were taken with a confocal (left panel) or using structured-illumination microscopy. Scale bar represents 10 μm for immunofluorescence. (b) Immunofluorescence images of MC1 (pathological tau - red), Map2 (neuronal dendrites - white) and bassoon (pre-synaptic – green) in rat primary neurons treated with a lentivirus overexpressing TauRD-YFP and rTg4510 tau seeds. Scale bar represents 10 μm. (c) Immunofluorescence images of MC1 (pathological tau - red), Map2 (neuronal dendrites - white) and bassoon (pre-synaptic – green) in rat primary neurons treated with a lentivirus overexpressing TauRD-YFP. Images were taken in the recipient chamber of a microfluidic device after treatment of the donor chamber with tau seeds extracted from rTg4510 mice. [file 40478_2021_1280_MOESM3_ESM.tif]

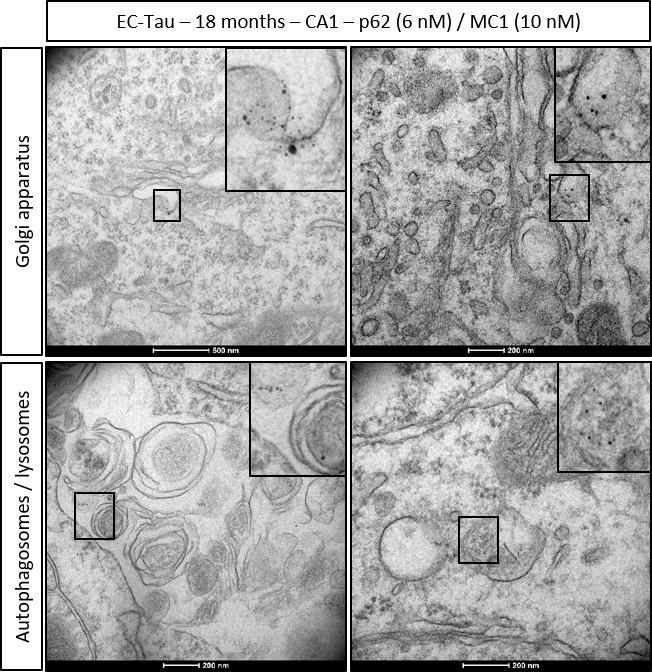

Supplement: Supplementary file 4 — Additional file 4: Fig. 4. p62 and MC1 colocalization with autophagy related structures. Images of p62/MC1 immunogold electron microscopy showing the Golgi apparatus (top panel), autophagosomes and lysosomes (bottom panel) all part of the cellular protein clearance system. Scale bar represents 200 μm. [file 40478_2021_1280_MOESM4_ESM.tif]

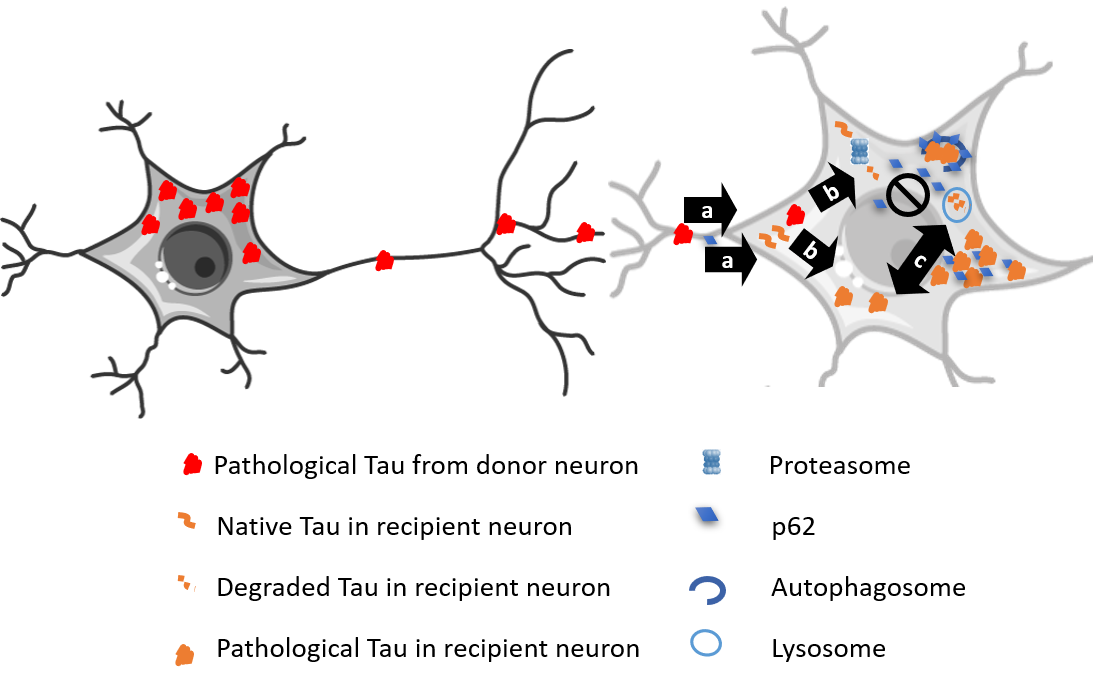

Supplement: Supplementary file 5 — Additional file 5: Fig. 5. Proposed model for the link between tau spread and inhibition of clearance mechanisms. (a) Transsynaptic spread of pathological tau from donor to recipient neurons (top) correlates with the spread of clearance deficits (bottom). (b) Pathological templating on native (bottom) tau & inhibition of clearance mechanisms, UPS and autophagy (top). (c) Representation of the proposed vicious circle between pathological tau accumulation and the inhibition of clearance mechanisms [file 40478_2021_1280_MOESM5_ESM.tif]
