# Supplementary material for: P62 accumulates through neuroanatomical circuits in response to tauopathy propagation
Source: Acta Neuropathol Commun. 2021 Nov 2;9:177. doi: 10.1186/s40478-021-01280-w (PMC8561893; doi:10.1186/s40478-021-01280-w)
Supplement: Supplementary file 6 — Additional file 6. Detailed statistics for quantifications in main and supplementary figures. [file 40478_2021_1280_MOESM6_ESM.docx]

**Supplementary data – statistics**

**Fig. 1f MC1 staining** **in seeded PS19 mice** – paired t-test region per region

| region | p-Value | t | df |
| --- | --- | --- | --- |
| DG | 0.0409 | 2.502 | 7 |
| CA3 | 0.0248 | 2.847 | 7 |
| CA1 | 0.0216 | 2.945 | 7 |
| Sub | 0.2773 | 1.178 | 7 |
| EC | 0.0143 | 3.237 | 7 |
| PRh | 0.7421 | 0.3424 | 7 |
| Front | 0.2105 | 1.378 | 7 |

**Fig. 1g P62 staining in seeded PS19 mice** – paired t-test region per region

| region | p-Value | t | df |
| --- | --- | --- | --- |
| DG | 0.1444 | 1.643 | 7 |
| CA3 | 0.0034 | 4.334 | 7 |
| CA1 | 0.0110 | 3.427 | 7 |
| Sub | 0.0011 | 5.315 | 7 |
| EC | 0.0010 | 5.395 | 7 |
| PRh | 0.0420 | 2.484 | 7 |
| Front | 0.1113 | 1.822 | 7 |

**Fig. 1h** **P62 correlation with MC1** **in seeded PS19 mice**

Spearman correlation R=0.5650 p=5.7*10^-6^

**Fig. 2f MC1 staining in EC-Tau -** one-way ANOVA followed by Tukey multiple comparison test for each region:

| region | F | p value | q Mild/Mod | p | q Mild/Sev | p | q Mod/Sev | p |
| --- | --- | --- | --- | --- | --- | --- | --- | --- |
| DG | 34.27 | <0.0001 | 8.971 | <0.0001 | 10.58 | <0.0001 | 1.487 | >0.05 |
| CA3 | 19.54 | <0.0001 | 6.379 | <0.01 | 8.223 | <0.001 | 1.708 | >0.05 |
| CA1 | 25.78 | <0.0001 | 7.852 | <0.001 | 9.126 | <0.0001 | 1.180 | >0.05 |
| Sub | 26.33 | <0.0001 | 7.937 | <0.001 | 9.221 | <0.0001 | 1.189 | >0.05 |
| EC | 32.62 | <0.0001 | 9.553 | <0.0001 | 9.673 | <0.0001 | 0.1119 | >0.05 |
| PRh | 24.12 | <0.0001 | 2.158 | >0.05 | 9.609 | <0.0001 | 6.898 | <0.001 |
| Front | 17.95 | =0.0001 | 2.496 | >0.05 | 8.401 | < 0.001 | 5.468 | <0.01 |

**Fig. 2g p62 staining in EC-Tau -** one-way ANOVA followed by Tukey multiple comparison test for each region:

| region | F | P value | q Mild/Mod | p | q Mild/Sev | p | q Mod/Sev | p |
| --- | --- | --- | --- | --- | --- | --- | --- | --- |
| DG | 15.65 | 0.0003 | 3.358 | >0.05 | 7.913 | <0.0001 | 4.216 | <0.05 |
| CA3 | 25.11 | < 0.0001 | 6.086 | <0.01 | 9.774 | <0.0001 | 3.414 | >0.05 |
| CA1 | 25.27 | < 0.0001 | 6.850 | <0.001 | 9.544 | <0.0001 | 2.495 | >0.05 |
| Sub | 28.81 | < 0.0001 | 6.340 | <0.01 | 10.52 | <0.0001 | 3.866 | <0.05 |
| EC | 32.76 | < 0.0001 | 7.592 | <0.001 | 10.95 | <0.0001 | 3.11 | >0.05 |
| Rh | 22.3 | < 0.0001 | 5.379 | <0.01 | 9.298 | <0.0001 | 3.628 | >0.05 |
| Front | 40.21 | = 0.0001 | 7.037 | <0.001 | 12.52 | <0.0001 | 5.079 | <0.01 |

**Fig. 2f MC1/p62 correlation in EC-Tau Spearman correlation**

R= 0.8108; p< 0.0001

**Fig. 3b p62 and MC1 in EC-Tau mice -** Two-way ANOVA subject matching 14 followed by Bonferroni multiple comparison test for each region:

| **Variation** | Interaction | | | Pathology level | | | Markers | | |
| --- | --- | --- | --- | --- | --- | --- | --- | --- | --- |
| region | Df | F |  | Df | F |  | Df | F |  |
| EC | 2 | 2.702 | 0.1018 | 2 | 41.92 | *** | 1 | 0.03701 | 0.8502 |
| Rh | 2 | 4.013 | 0.04919 | 2 | 28.58 | *** | 1 | 13.73 | 0.0024 |
| Front | 2 | 39.41 | *** | 2 | 40.06 | *** | 1 | 160.5 | *** |

| **Bonferroni** | Mild | | Moderate | | Severe | |
| --- | --- | --- | --- | --- | --- | --- |
| region | t | p-Value | t | p-Value | t | p-Value |
| EC | 0.7885 | ns | 1.958 | ns | 0.9746 | ns |
| Rh | 1.425 | ns | 4.287 | ** | 0.6140 | ns |
| Front | 1.075 | ns | 7.635 | *** | 12.33 | *** |

**Fig. 3d seeding assay in EC-Tau -** one-way ANOVA followed by Tukey multiple comparison test for each region:

| region | F | P value | q Mild/Mod | p | q Mild/Sev | p | q Mod/Sev | p |
| --- | --- | --- | --- | --- | --- | --- | --- | --- |
| Hippo | 20.90 | 0.0020 | 3.533 | <0.05 | 6.445 | <0.01 | 1.960 | >0.05 |
| EC | 27.50 | 0.0010 | 6.075 | <0.01 | 6.640 | <0.01 | 0.5471 | >0.05 |
| Rh | 11.76 | 0.0084 | 2.343 | >0.05 | 4.850 | <0.01 | 1.808 | >0.05 |
| Front | 3.129 | 0.1173 | 0.6136 | >0.05 | 2.423 | >0.05 | 1.490 | >0.05 |

**Fig. 5 proteasome inhibition in primary neurons -** one-way ANOVA followed by Bonferroni multiple comparison test – comparison to DMSO:

**Fig. 5e**: MG132 F(5, 14) = 31.03 ; p<0.0001

| Dose | t | p |
| --- | --- | --- |
| 10 nM | 0.7302 | >0.05 |
| 250 nM | 3.932 | <0.01 |
| 500 nM | 6.289 | <0.0001 |
| 1000 nM | 6.710 | <0.0001 |

**Fig. 5f**: Epoxomycin F(4, 13) = 32.51 ; p<0.0001

| Dose | t | p |
| --- | --- | --- |
| 1 nM | 1.062 | >0.05 |
| 10 nM | 4.547 | <0.01 |
| 20 nM | 6.905 | <0.0001 |

**Supplementary Fig. 2 EC-Tau NeuN** Two-way ANOVA followed by Bonferroni multiple comparison test for each region

| **Variation** | Interaction | | Genotye | | Age | |
| --- | --- | --- | --- | --- | --- | --- |
| region | Df | F | Df | F | Df | F |
| PaS | 2 | 31.68 | 1 | 79.19 | 2 | 17.93 |
| MEC | 2 | 6.137 | 1 | 11.26 | 2 | 23.76 |
| LEC | 2 | 1.155 | 1 | 2.843 | 2 | 2.009 |
| DG | 2 | 1.900 | 1 | 0.8597 | 2 | 0.9530 |
| CA3 | 2 | 1.766 | 1 | 0.2999 | 2 | 2.265 |
| CA1 | 2 | 4.700 | 1 | 0.6761 | 2 | 1.544 |
| Sub | 2 | 2.892 | 1 | 0.1149 | 2 | 0.7854 |
| Rh | 2 | 1.205 | 1 | 1.816 | 2 | 0.3534 |
| Front | 2 | 0.08034 | 1 | 0.02446 | 2 | 0.1889 |

| **Bonferroni** | Young | | Medium | | Old | |
| --- | --- | --- | --- | --- | --- | --- |
| region | t | p-Value | t | p-Value | t | p-Value |
| PaS | 0 | ns | 4.253 | *** | 10.94 | *** |
| MEC | 0 | ns | 1.074 | ns | 4.674 | *** |
| LEC | 0 | ns | 2.053 | ns | 0.7837 | ns |
| DG | 0 | ns | 2.055 | ns | 0.5267 | ns |
| CA3 | 0 | ns | 1.739 | ns | 0.8547 | ns |
| CA1 | 0 | ns | 2.785 | * | 1.463 | ns |
| Sub | 0.7885 | ns | 1.958 | ns | 0.9746 | ns |
| Rh | 1.425 | ns | 4.287 | ** | 0.6140 | ns |
| Front | 1.075 | ns | 7.635 | *** | 12.33 | *** |

**Supplementary Fig. 2 EC-Tau Cd68** Two-way ANOVA followed by Bonferroni multiple comparison test for each region

| **Variation** | Interaction | | Genotye | | Age | |
| --- | --- | --- | --- | --- | --- | --- |
| region | Df | F | Df | F | Df | F |
| PaS | 2 | 24.70 | 1 | 90.91 | 2 | 42.58 |
| MEC | 2 | 11.14 | 1 | 48.36 | 2 | 22.40 |
| LEC | 2 | 14.54 | 1 | 47.33 | 2 | 44.24 |
| DG | 2 | 1.265 | 1 | 0.2322 | 2 | 21.66 |
| CA3 | 2 | 6.326 | 1 | 7.706 | 2 | 18.08 |
| CA1 | 2 | 4.134 | 1 | 21.77 | 2 | 28.43 |
| Sub | 2 | 8.123 | 1 | 43.92 | 2 | 48.92 |
| Rh | 2 | 1.244 | 1 | 3.532 | 2 | 41.38 |
| Front | 2 | 1.532 | 1 | 4.872 | 2 | 36.74 |

| **Bonferroni** | Young | | Medium | | Old | |
| --- | --- | --- | --- | --- | --- | --- |
| region | t | p-Value | t | p-Value | t | p-Value |
| PaS | 0.6338 | ns | 5.363 | *** | 10.94 | *** |
| MEC | 0.3891 | ns | 4.846 | *** | 6.608 | *** |
| LEC | 0.3283 | ns | 3.672 | ** | 7.752 | *** |
| DG | 0.3974 | ns | 1.320 | ns | 0.9116 | ns |
| CA3 | 0.02289 | ns | 0.2769 | ns | 4.478 | *** |
| CA1 | 0.7943 | ns | 2.484 | ns | 4.721 | *** |
| Sub | 0.8793 | ns | 4.131 | *** | 6.317 | *** |
| Rh | 0.5866 | ns | 0.3093 | ns | 2.363 | ns |
| Front | 2.465 | ns | 1.463 | ns | 0.04892 | ns |
